# The learning environment of four undergraduate health professional schools: Lessons learned

**DOI:** 10.12669/pjms.35.3.712

**Published:** 2019

**Authors:** Farhana Irfan, Eiad Al Faris, Nasr Al Maflehi, Syed Irfan Karim, Gominda Ponnamperuma, Hussain Saad, Abdullah MA Ahmed

**Affiliations:** 1 *Farhana Irfan, FRCGP., College of Medicine, Department of Family and Community Medicine, King Saud University Chair for Medical Education Research and Development, King Saud University, Riyadh, Saudi Arabia*; 2 *Eiad Al Faris, MMed., College of Medicine, Department of Family and Community Medicine, King Saud University Chair for Medical Education Research and Development, King Saud University, Riyadh, Saudi Arabia*; 3 *Nasr Al Maflehi, MSc. College of Dentistry, King Saud University, Riyadh, Saudi Arabia*; 4 *Syed Irfan Karim, FRCGP., College of Medicine, Department of Family and Community Medicine, King Saud University Chair for Medical Education Research and Development,*; 5 *Gominda Ponnamperuma, PhD. Centre for Medical Education, National University of Singapore, Singapore*; 6 *Hussain Saad, MRCP., College of Medicine, Department of Family and Community Medicine, King Saud University Chair for Medical Education Research and Development, King Saud University, Riyadh, Saudi Arabia*; 7 *Abdullah MA Ahmed, BSc., College of Medicine, Department of Family and Community Medicine, King Saud University Chair for Medical Education Research and Development, King Saud University, Riyadh, Saudi Arabia*

**Keywords:** Curriculum, DREEM, Health professional students, Learning environment, Student perceptions

## Abstract

**Background and Objectives:**

Learning is an interplay between cognition and environmental factors. Any learning environment, that fulfills the intrinsic and extrinsic needs of the students will probably lead to better and more promising learning outcomes. This study aimed to investigate the student perceptions of Learning Environment (LE) in four health schools of a large university and compare between schools, years of study, and gender.

**Methods:**

Dundee Ready Education Environment Measure (DREEM) questionnaire and a socio-demographic questionnaire were completed by 1185 undergraduate students enrolled in the school of Medicine, Dentistry, Nursing and Applied Medical Sciences (AMS) of a large university during the academic year 2012-2013. Chi-square test was used to compare categorical variables. Independent student t-test or ANOVA (with Tukey post-hoc test) was used for continuous variables at a significance level of p≤0.05.

**Results:**

The mean total DREEM score was 89.23±33.3. The total DREEM mean scores for Dentistry (120.54±23.45) and Medicine (110.72±19.33) were higher compared with AMS (63.48±21.36) and Nursing (57.48±22.80) (p=0.000) (Post hoc Tukey p=0.000). First year students gave significantly higher positive perceptions ratings than the rest of the years (p=0.000). Total scores were significantly higher for male (92.78±33.86) than female students (84.70±32.25) p=0.000.

**Conclusion:**

The LE significantly differed by year and gender. The students from non-integrated curricula (nursing and AMS) perceived the LE less positively than their integrated curriculum counterparts (medicine and dentistry). A qualitative study is needed to investigate the variation in the perception of LE among these groups.

## INTRODUCTION

Learning Environment (LE) refers to the social, psychological, and pedagogical contexts in which learning occurs.^1^ The LE plays a central role in learning and contributes positively to students’ achievement, satisfaction, success and is one of the essential factors in enriching students’ learning.^2^,^3^ The students are the customers of the learning process; their feedback therefore plays a pivotal role for the success of any educational climate.^4^ An emphasis on monitoring the learning experience and effort to improve it by regular monitoring and feedback from students is an essential component in establishing the value of the learning experience for students.^5^ The students’ perceptions of their current LE is found to be a stronger predictor of learning outcomes at university than to their prior achievement at school.^6^ From an educational theory perspective, it is better to determine the morphological characteristics of LE such as pedagogical philosophy, curriculum design and social climate. The latter has sometimes been looked at as a ‘peripheral’ factor in the provision of quality education. Although it might seem that the concept of LE is rather intangible, its effects are influential and real, affecting students’ achievements, attitudes and well- being.^4^,^7^

International trends in education show a shift from the traditional non-integrated, teacher-centered approach to an integrated, student-centered approach.^3^,^8^ Studies looking at the LE of different health professional (HP) schools worldwide have shown a trend of higher total scores in the student-centered schools compared with teacher centered (traditional) schools.^4^,^9^-^12^ A study in a large medical school in Saudi Arabia, which transitioned from a traditional to a system-based curriculum found that type of the curriculum has a direct impact on LE and students’ wellbeing for both genders.^12^

Various methodologies have been utilized to investigate the LE.^13^ The Dundee Ready Education Environment Measure (DREEM) is one of the most widely used instruments designed to assess the LE and has been used in several undergraduate courses for health professionals worldwide, including in Saudi Arabia.^12^,^14^,^15^ Utilizing DREEM among HP students would provide a snapshot of the students’ perception of their LE.

LE studies have focused separately on single HP schools; mainly on the medical and dental schools. To the best of our knowledge, none of the studies has addressed LE with disciplines such as nursing, dentistry, medicine and applied medical sciences in a single study locally or internationally.

Little is known how their students differ in their LE perception. The current study was carried out in a large University that has both integrated and non-integrated curriculum, providing an ideal opportunity to study and compare the four health professional schools together.

The findings from this study can be used as a reference point for future studies involving HP students. It would provide the patterns and trends that exist among schools, study years and gender.^2^,^16^ The main objectives of the current study were to

1. Investigate the student perception of LE in four health schools of a large university and
2. Identify the associations between the LE and some sociodemographic factors such as school of study, years of study and gender.

## METHODS

This cross sectional descriptive study was conducted at King Saud University (KSU), which offers a six-year baccalaureate degree program in schools of Medicine and Dentistry. These schools revised and reformed its curriculum to be integrated, system oriented, problem based, student centered and community oriented. The medical degree is followed by a one-year internship. The Applied Medical Sciences (AMS) and nursing schools offer four-year bachelor degree programs followed by a one-year internship. The programs Leading to B.Sc Degree in AMS are Biomedical Technology, Clinical Laboratory Sciences, Community Health Sciences: (Clinical Nutrition, Health Education, Health Services Administration, Medical Records Administration) Dental Health: (Dental Hygiene, Dental Technology), Optometry, Radiological Sciences, Rehabilitation Sciences: (Physical Therapy, Speech and Hearing Therapy, Respiration Therapy, Functional Therapy) and M. Sc. Degree in Clinical Laboratory Sciences, Clinical Nutrition, Physical Therapy and Optometry and vision Sciences. These schools follow a non-integrated and teacher-centered curriculum at present. KSU operates on a separate gender basis.

### Sampling

A cross-sectional, descriptive study design was used. The estimated sample size was 1500 students. A stratified random sampling technique using the proportional allocation method was used.to determine the number of students based on their school, gender and the year of study.

### Participants

Participants included students enrolled in four undergraduate programs (Medicine, Dentistry, Nursing, and AMS) offered at the King Saud University (KSU), Riyadh, Saudi Arabia in the academic year 2012-2013.

### Instruments

A short demographic questionnaire was developed to collect the participant’s demographic information such as age, sex, school and the year of study.

The DREEM inventory was used to measure the perception of the LE. It comprises 50 statements /items relating to a range of topics directly relevant to LE on a 5-point Likert scale. The items of the inventory were scored 4 for Strongly Agree (SA), 3 for Agree (A), 2 for Uncertain (U), 1 for Disagree (D) and 0 for Strongly Disagree (SD). However, 9 negatively worded were scored in the reverse order. The inventory has a maximum score of 200 which indicates the ideal LE.^17^ The interpretation of the DREEM total score is 0-50 (very poor), 51-100 (plenty of problems), 101-150 (more positive than negative) and 151-200 (excellent).^18^

The DREEM consists of five subscales, namely students’ perceptions of learning (SPL), students’ perceptions of teachers (SPT), students’ academic self-perceptions (SASP), students’ perception of atmosphere (SPA) and students’ social perceptions (SSP)^17^. Scores less than 25 for SPL, 23 SPT, 17 SASP, 25 SPA,15 SSP are considered low, with a lot of problems.

### Data collection

The DREEM and the demographic questionnaires along with the covering letter were distributed to the study population. The students were informed about the aims of the study, anonymity and confidentiality. The participation was voluntary.

For quality assurance of the data collection, a group of students (named here as team leaders) were selected and trained based on the recommendations of their schools’ vice deans and school administrators.

For each school and study year, team leaders were assigned with the ratio of one leader for each 25- 30 students. Team leaders were awarded some financial incentives. In person inventories were given to the students and data were gathered through selected team leaders. Students returned the completed questionnaires either immediately or one day later.

### Ethical considerations

The current study was approved by the Institutional Review Board of the School of Medicine; King Saud University (reference no. 11/3106/IRB). Participants were given an explanatory statement detailing the study aims and their consent to take part in the study was inferred by their completion of the questionnaire. All the selected respondents were given assurance of confidentiality.

### Statistical analysis

All the collected data were entered into the statistical package for social sciences (SPSS) version 19. Means and standard deviations were calculated for DREEM total and subscale for the entire sample as well as for the schools. For dichotomous variables comparisons of total and subscale DREEM means were carried out using independent t tests. Analysis of variance (ANOVA) was used with variables more than two factors. Where ANOVA indicated a significant difference in group means, Tukey’s HSD (adjusted for multiple comparisons) was used to make post hoc comparisons to find out which specific groups means are different and a *p* value less than 0.05 was considered significant.

## RESULTS

A total number of 1186 students (response rate of 79%) filled out the DREEM inventory. The highest number of students were from the school of AMS (39%; n=461), Medicine (37%; n=435) followed by dentistry (16%; n=186) and least proportion was from school of nursing (9%; n=104). More than half (56%) of the students were males. The age ranged from 18-34 years. The mean age of students was 21.34±1.58 years.

The descriptive statistics associated with the students DREEM scores across the four HP student groups are reported in Table-I. The mean total DREEM scores varied significantly between schools (p=0.000). The scores were lower for AMS 63.48 (21.36) and nursing 57.48 (22.80), compared with dentistry 120.54 (23.45) and medicine 110.72 (19.33). (Table-I). To evaluate the differences further between the four schools, the statistically significant ANOVA findings were followed up by the Tukey HSD test. Post hoc comparisons indicated the mean score for the medicine and dentistry students was significantly higher than the total DREEM scores of the nursing and AMS students. However, the nursing did not significantly differ from the AMS. Similarly, all subscale scores varied significantly between schools (p=0.000) (Table-II).

**Table-I:** Mean (SD) subscale and total DREEM scores for HP students.

	MED* Mean (SD)	DENT* Mean (SD)	AMS* Mean (SD)	NUR* Mean (SD)	Total (across schools) Mean(SD)	P-value	Tukey <0.05
SPL	25.71 (6.69)	28.20 (7.089)	13.86 (7.26)	11.82 (6.25)	20.28(9.54)	0.000	DENT- NUR, AMS, MED-NUR, AMS
SPT	23.60 (4.67)	26.49 (5.57)	15.22 (5.71)	13.73 (6.38)	19.93(7.26)	0.000	DENT-NUR, AMS, MED-NUR, AMS
SAP	19.54 (4.54)	21.00 (4.6)	8.22 (4.69)	7.64 (4.96)	14.33(7.55)	0.000	DENT-NUR, AMS, MED-NUR, AMS
SPA	24.87 (6.397)	27.06 (7.294)	15.57 (6.674)	14.91 (7.059)	20.73(8.414)	0.000	DENT-NUR, AMS, MED-NUR, AMS
SSP	16.42 (3.714)	17.55 (4.015)	10.21 (3.878)	9.19 (3.433)	13.55 (5.095)	0.000	DENT-NUR, AMS, MED-NUR, AMS
Total	110.72 (19.33)	120.54 (23.45)	63.48 (21.36)	57.48 (22.80)	89.23(33.39)	0.000	DENT-NUR, AMS, MED-NUR, AMS

DENT*: Dental, MED*: Medicine, NUR*: Nursing, AMS*: Applied medical science, **SPL:** Students’ perceptions of learning, **SPT:** Students’ perceptions of teachers, **SAP:** Students’ academic self-perceptions, **SPA:** Students’ perception of atmosphere, **SSP:** Students’ social perceptions.

**Table-II:** Mean (SD) Students’ score for all the DREEM items.

Sr #	Item	N = 1186 Mean (SD)
1	I am encouraged to participate in class	1.70(1.37)
2	The teachers are knowledgeable	1.97(1.43)
3	There is a good support system for students who get stressed	1.45(1.26)
4	I am too tired to enjoy the course	2.02(1.36)
5	Learning strategies which worked for me before continue to work for me now	1.55(1.30)
6	The teachers are patients with patients	1.76(1.25)
7	the teaching is often stimulating	1.57(1.28)
8	The teachers ridicule the students	1.94(1.39)
9	The teachers are authoritarian	1.82(1.32)
10	I am confident about my passing this year	2.07(1.44)
11	The atmosphere is relaxed during the ward teaching	1.59(1.29)
12	The school is well timetabled	1.83(1.44)
13	The teaching is student centered	1.62(1.31)
14	I am rarely bored in this course	1.53(1.39)
15	I have good friends in this school	2.40(1.44)
16	The teaching helps to develop my competences	1.82(1.33)
17	Cheating is a problem in this school	2.02(1.42)
18	The teachers have good communication skills with patients	1.75(1.29)
19	My social life is good	2.06(1.42)
20	The teaching is well focused	1.62(1.32)
21	I feel I am being well prepared for my profession	1.70(1.32)
22	The teaching helps to develop my confidence	1.64(1.33)
23	The atmosphere is relaxed during lectures	1.72(1.34)
24	The teaching time is put to good use	1.53(1.28)
25	The teaching over-emphasized factual learning	1.91(1.31)
26	Last year work has been a good preparation for this year work	1.71(1.37)
27	I am able to memorize all I need	1.55(1.32)
28	I seldom feel lonely	1.82(1.43)
29	The teachers are good at providing feedback to students	1.56(1.30)
30	There are opportunities for me to develop interpersonal skills	1.74(1.38)
31	I have learned a lot about empathy in my profession	2.00(1.43)
32	The teachers provide constructive criticism here	1.65(1.30)
33	I feel comfortable in class socially	1.82(1.43)
34	The atmosphere is relaxed during seminars / tutorials	1.80(1.40)
35	I find the experience disappointing	1.83(1.39)
36	I am able to concentrate well	1.60(1.36)
37	The teachers give clear examples	1.82(1.37)
38	I am clear about the learning objectives of the course	1.71(1.39)
39	The teachers get angry in class	1.88(1.31)
40	The teachers are well prepared for their classes	1.89(1.41)
41	My problem solving skills are being well developed here	1.86(1.38)
42	The enjoyment outweighs the stress of the course	1.55(1.34)
43	The atmosphere motivates me as a learner	1.62(1.29)
44	The teaching encourages me to be an active learner	1.65(1.28)
45	Much of what I have o learn seems relevant to a career in health care	1.92(1.37)
46	My accommodation is pleasant	2.31(1.43)
47	Long term learning is emphasized over short term learning	1.69(1.36)
48	The teaching is too teacher centered	1.93(1.28)
49	I feel able to ask all the questions I want	1.73(1.37)
50	The students irritate the teachers	2.00(1.32)

The most negative ratings given by the students were for items “There is a good support system for students who get stressed”, “Learning strategies which worked for me before continue to work for me now”, “The teaching is often stimulating”, “The atmosphere is relaxed during the ward teaching”, “I am rarely bored in this course”, “The teaching time is put to good use”, “I am able to memorize all I need”, “The teachers are good at providing feedback to students”, “The enjoyment outweighs the stress of the course”. (Table-II).

Total DREEM scores were significantly higher for male students (92.37; SD 33.86) than their female counterparts (84.29; SD 32.25) (p=0.000). Similarly, all subscale scores varied significantly between the two genders (p=0.000) (Table-III).

**Table-III:** Mean (SD) subscale and total DREEM scores for HP students by gender.

	Students’ perception of learning	Students’ perception of teachers SPT	Students’ academic self-perceptions SAP	Students’ perception of atmosphere SPA	students’ social self-perceptions SSP	Total
Male(667)	21.23 (9.606)	20.32 (7.480)	15.23 (7.440)	21.68 (8.642)	13.9 (5.158)	92.37 (33.86)
Female(518)	19.08 (9.337)	19.42 (6.957)	13.18 (7.54)	19.52 (7.959)	13.09 (4.982)	84.29 (32.25)
*p*-value	0.000	0.036	0.000	0.000	0.006	0.000

Significant differences in the total DREEM scores were found between the study years (p=0.000). First year students gave significantly higher ratings than the rest of the years for all the five subscales (Table-IV).

**Table IV:** Mean subscale DREEM scores for HP students by year of study (post hoc=TUKEY) (p=0.000).

Mean DREEM score (SD)							P-value	Tukey <0.05

Academic year	1 N=181	2 N=249	3 N=262	4 N=319	5 N=168	Total N=1179		

Domain								
students’ perception of learning	25.22 (8.23)	20.11 (9.59)	18.21 (9.16)	20.07 (10.00)	19.01 (8.77)	20.30 (9.55)	0.000	1-2,3,4,5
students’ perception of teachers	21.78 (5.44)	19.03 (6.51)	19.83 (7.44)	20.03 (8.23)	19.45 (7.49)	19.96 (7.26)	0.002	1-2,3,5
students’ academic self-perceptions	17.24 (6.12)	14.50 (7.68)	12.93 (7.29)	13.77 (7.84)	14.26 (7.75)	14.34 (7.54)	0.000	1-2,3,4,5
students’ perception of atmosphere	24.64 (7.36)	19.92 (8.01)	19.36 (7.75)	20.76 (9.11)	19.88 (8.52)	20.74 (8.42)	0.000	1-2,3,4,5
students’ social self-perceptions	16.27 (4.74)	13.27 (4.74)	12.77 (5.13)	13.37 (5.39)	12.71 (4.38)	13.56 (5.10)	0.000	1-2,3,4,5

Total	105.14 (4.12)	86.83 (3.23)	83.10 (3.49)	87.99 (3.69)	85.29 (3.32)	89.0 (3.51)	0.000	1-2,3,4,5,

## DISCUSSION

The current study found a striking difference in the LE between the schools of dentistry and medicine on one hand and the AMS and nursing on the other hand. LE was perceived to have many problems in the schools of nursing and AMS. While medical and dental students had positive perception of their LE.

Overall, the low mean total DREEM scores among AMS and nursing students are alarming and indicate that there are more negative than positive points in the LE. These values for AMS and nursing students are below most of the previous studies findings.^19^,^20^

A move towards the development of an effective student support system to provide emotional support, encouragement and necessary guidance and a strong faculty development program to train the teachers to improve their teaching skills is needed.

Studies in other parts of the world suggest a more positive LE mostly after change in curriculum and making some reform. For instance, a school of Medicine in Aruba (an island in the southern Caribbean Sea), following an integrated organ system based curriculum has reported a score of 131/200.^21^ Studies from the UK and Chile have reported a score of 139 and 127.5 in schools following innovative curricula.^9^,^10^ Similarly, positive LE perceptions were found among the medical students following the new integrated curriculum in the current study, with a score of 110. It is interesting to note the low DREEM score among KSU medical students few years ago as reported by previous two studies,^4^,^14^ when teaching was traditional and teacher centered, has improved significantly in the current study. Few studies have reported even higher results than the present study.^11^,^20^,^22^ In a different country and within the field of dentistry, Ali K et al.^23^ detected a value of 143, confirming a more positive LE. This indicates that there is room for improvement if the instructional methods are made more relaxed, practical and enjoyable, as done previously in the school of medicine with positive results.^12^

In line with the previous studies, DREEM scores were significantly higher for male than female students.^24^ A possible reason for this gender-based difference could be the differences in the learning experience. In the field of education, it is believed that girls and boys have different learning needs^25^ and deeply embedded gender stereotypes can also cause faculty to respond differently to male and female students. Another possibility could be the availability of a less efficient support system for the female students. The study design does not allow us to provide explanation to these possibilities. An in depth, qualitative study may explain the variations of the DREEM scores for different health schools.

There was a decline in the students’ perception of all domains from the first to the fifth study years. This is consistent with previous study findings.^4^,^14^ A plausible explanation is that the students were enthusiastic and had high hopes and optimism about their schools initially, and have not experienced the stressful aspects of the environment. And they were disappointed in later years due to curriculum overload, stress, weak support system, little feedback and less than ideal teaching methods. This deserves further exploration. The highest decline in the scores was observed in the third year which coincides with the commencement of clinical training. This trend could be because the students were exposed to a new challenging clinical environment, a period when they deal with patients and face adjustment problems. The finding is in line with another study conducted in the Faculty of Nursing, International Islamic University Malaysia.^20^

### Limitations and recommendations

DREEM in general exposes the ‘tip of the iceberg’ and indicates the students’ areas of concern and does not provide explanations and reasons for these concerns. Therefore, exploratory qualitative studies are needed to fill this gap. The results cannot be generalized beyond the study setting as the study was conducted in one university.

The unique nature of the study in terms of addressing the LE in four health schools of the same university makes it a reference for future studies involving HP students. In addition, is there a similar pattern among the different health professions schools in other universities? Such questions are important to be assessed and need to be addressed in future research.

## CONCLUSION

Total scores for LE were higher for male than female students and were highest for first year students and lowest for third year students. The curriculum type of the school is a major determinant of the learning environment as perceived by students. The students from non-integrated curricula (nursing and AMS) perceived the LE less positively than their integrated curriculum counterparts (medicine and dentistry). A qualitative study is needed to explore further the associated factors and explanations for the variation in the perception of LE among these groups.
